# Long non‐coding RNA MFAT1 promotes skeletal muscle fibrosis by modulating the miR‐135a‐5p‐Tgfbr2/Smad4 axis as a ceRNA

**DOI:** 10.1111/jcmm.16508

**Published:** 2021-04-09

**Authors:** Jinrong Lin, Xiaobao Yang, Shaohua Liu, Zhiwen Luo, Qingyan Chen, Yaying Sun, Zheci Ding, Jiwu Chen

**Affiliations:** ^1^ Department of Sports Medicine Huashan Hospital Fudan University Shanghai China; ^2^ Department of Medical Laboratory Science Ruijin Hospital School of Medicine Shanghai Jiao Tong University Shanghai China; ^3^ Biology Department Boston University Boston MA USA

**Keywords:** long noncoding RNA, miR‐135a‐5p, skeletal muscle fibrosis, TGFβ pathway

## Abstract

Fibrosis after skeletal muscle injury is common in sports and can cause irreversible damage to the biomechanical properties of skeletal muscle. Long non‐coding RNAs (lncRNAs) have been validated to act as important modulators in the fibrosis of various organs. Here, we reported a novel lncRNA (the skeletal muscle fibrosis‐associated transcript 1, lnc‐MFAT1), which was highly expressed in skeletal muscle fibrosis. We demonstrate that lnc‐MFAT1 knockdown can reduce TGFβ‐induced fibrosis in vitro and attenuate skeletal muscle fibrosis after acute contusion in mice. Further study showed that lnc‐MFAT1 acted as a competitive endogenous RNA of miR‐135a‐5p. Besides, the miR‐135a‐5p inhibition obviously promoted TGFβ‐induced fibrosis in vitro via enhancing its target genes Tgfbr2/Smad4. Moreover, we discovered that lnc‐MFAT1 regulates Tgfbr2/Smad4 expression by sponging miR‐135a‐5p to exert competing endogenous RNA function, resulting in TGFβ pathway activation. In conclusion, our study identified a crucial role of lnc‐MFAT1‐miR‐135a‐Tgfbr2/Smad4 axis in skeletal muscle fibrosis, providing a promising treatment option against skeletal muscle fibrosis.

## 1 INTRODUCTION

Skeletal muscle injury is common in sports, with a reported incidence varying from 10% to 55%.^1^, ^2^, ^3^ Injured skeletal muscle cannot fully recover its biomechanical properties because of fibrosis, which is the excessive production and deposition of extracellular matrix (ECM). The formation of fibrosis hinders muscle regeneration, impairs muscle contraction and distorts dynamic properties, leading to higher probability of re‐injury.^4^ Therefore, intervention against fibrosis is critical to improve the post‐operative healing of skeletal muscle.

Transforming growth factor β (TGF‐β) is one of the main signalling molecules initiating fibrosis.^5^ Previous studies from our laboratory demonstrated that miRNA could modulate TGF‐β/Smad signalling pathway in skeletal muscle fibrosis.^6^, ^7^ Recently, other studies reported the lncRNAs could regulate the TGF‐β/Smad pathway,^8^, ^9^ and they hold essential roles in the development and progression of fibrosis in various organs, including liver, lung, heart, kidney and skeletal muscle.^10^, ^11^

As a competing endogenous RNA (ceRNA), lncRNAs typically participated in post‐transcriptional regulation by interacting with miRNAs or mRNAs in the cytoplasm.^12^, ^13^ However, the function and involvement of lncRNA‐miRNA‐TGF‐β/Smad pathway crosstalk remains unclear and might be a key point in finding the potential therapeutic targets against skeletal muscle fibrosis.

In current study, we first reported a novel lncRNA (the skeletal muscle fibrosis‐associated transcript 1, lnc‐MFAT1), which was markedly up‐regulated in a skeletal muscle fibrosis mouse model and correlated with poor recovery. Through a series of in vitro and in vivo experiments, we demonstrated that silencing lnc‐MFAT1 can alleviated skeletal muscle fibrosis after acute contusion. lnc‐MFAT1 functioned as a ceRNA of miR‐135a‐5p (miR‐135a) and subsequently regulated Tgfbr2/Smad4 expression. The present study identified a crucial role of lnc‐MFAT1‐miR‐135a‐Tgfbr2/Smad4 axis in skeletal muscle fibrosis, providing a promising treatment option against skeletal muscle fibrosis.

## 2 MATERIALS AND METHODS

### 2.1 Animal experiments

C57BL/6J mice aged 10 weeks were purchased from Vital Co. and were maintained in a 12‐h light/dark cycle with free access to food and water. All animal experiments procedures were carried out following the technical guidelines and approved by the Animal Care and Use Committee of Fudan University. The acute contusion‐induced mouse skeletal muscle fibrosis model of the right tibialis anterior (TA) was established following the previous method.^7^ Mice were anaesthetized by intraperitoneal injection of 1% sodium pentobarbital (0.5 mL/100 g) and immobilized with its right hindlimb taped to a plate. After that the TA was exposed and hit by a stainless‐steel ball (2 cm in diameter, 15 g in weigh) dropping from a height of 1 m. The left TA served as control.

### 2.2 In vivo adeno‐associated virus (AAV) particle administration

Thirty‐six skeletal muscle fibrosis mice models were randomly assigned into three groups: acute contusion without injection (n = 12), acute contusion combined with injection of AAV‐NC (n = 12) and acute contusion in combined with injection of AAV‐MFAT1 (n = 12). Following established protocols with minor modifications, aliquots of 0.1 mL (1 × 10^12^ AAV particles containing either a lnc‐MFAT1 or a null vector) were injected around the circumference of one side TA muscles with a 27‐gauge needle. A total of 15 μL of viral preparation were injected into each muscle. Twenty‐eight days later, all of mice were killed. TA was collected and analysed by real‐time RT‐PCR, Western blot, Masson staining and immunohistochemical analysis to determine the mRNA and protein expression of fibrosis indicators.

### 2.3 Cell culture and antibodies

C2C12 cells and HEK293T were obtained from the cell bank of the Chinese Academy of Sciences (Shanghai, China). Both cells were maintained in DMEM (HyClone) containing 10% foetal bovine serum (FBS; Gibco‐BRL), penicillin (100 U/mL) and streptomycin (100 μg/mL). All cells were cultured in humid atmosphere containing 5% CO_2_ at 37°C. Primary antibodies for Collagen1 (Col1), Vimentin (VIM), α‐SMA, TGFBR2, Smad4 and GAPDH were purchased (Table S2). Donkey anti‐rabbit IgG and donkey anti‐mouse IgG served as secondary antibodies for immunohistochemical analysis, whereas goat anti‐rabbit IgG for Western blot (Table S2).

### 2.4 Cell transfection

The transfection was carried out using the Lipofectamine 3000 Reagent (Invitrogen) following the manufacturer's protocol. The small interfering RNAs against lnc‐MFAT1 (si‐MFAT1), negative control (si‐NC) and the pcDNA3.1 vector targeting lnc‐MFAT1 were all acquired from Sangon Biotech. To knockdown or overexpress miR‐135a, miR‐135a mimics, miR‐135a inhibitors and its negative controls were purchased from Sangon Biotech and were transfected into C2C12 cells. The sequences are listed in Table S2.

### 2.5 Western blot

Total proteins were prepared in RIPA buffer (Beyotime) supplemented with protease and phosphatase inhibitor cocktail (Pierce, Thermo Fisher Scientific). Total protein quantity was determined using BCA protein assay (BCA Protein Assay Kit; Pierce Thermo Fisher Scientific). Then, samples were normalized and separated by 10% SDS‐polyacrylamide gel electrophoresis (SDS‐PAGE) and transferred onto polyvinylidene fluoride (PVDF) membranes (Millipore). After that, the membrane was blocked with 5% skim milk in 1×TBST for 1 hour at room temperature and then immunoblotted in primary antibodies solution at 4°C overnight. After that, the membranes were washed in 1×TBST and incubated with goat anti‐rabbit IgG for 1 hour at room temperature. The proteins were visualized using ECL reagent (Beyotime) and imaged using Tanon‐5200 Multi Fluorescence imager. Antibodies used for Western blot are listed in supplemental Table S2.

### 2.6 Quantitative real‐time PCR (qRT‐PCR) analysis

Total RNA from cells was isolated using TRIzol reagent (Invitrogen). Afterwards, cDNA was synthesized using Advantage RT‐for‐PCR Kit (TaKaRa). qRT‐PCR was implemented with SYBR Green I Master (Roche) on a 7500 Real‐Time PCR System (Applied Biosystems).

GAPDH and U6 were used as endogenous control for lncRNA/mRNA and miRNA, respectively.

Expression fold change was calculated using the 2−ΔΔCt method. All primer sequences are listed in supplemental Table S1.

### 2.7 Cytoplasmic and nuclear protein fractionation

Cytoplasmic and nuclear RNA isolation were carried out with PARIS™ Kit (Invitrogen) according to the manufacturer's instruction. Then, qRT‐PCR was used for measurement of nuclear and cytoplasmic RNA. β‐Actin and U6 were used as cytoplasmic and nuclear controls, respectively.

### 2.8 Dual‐Luciferase reporter assay

The putative miR‐135a binding sites in lnc‐MFAT1, Tgfbr2‐3ʹ‐UTR or Smad4‐3ʹ‐UTR and the mutant binding sites were constructed and cloned to the pSI‐Check2 luciferase vector (Hanbio), immediately downstream of the luciferase gene. HEK293T cells were cotransfected with the pSI‐Check2 reporter plasmids and the miR‐135a‐5p mimics or miR‐135a‐5p inhibitor, respectively. Twenty‐four hours after transfection, the fluorescence intensity was detected with the Dual‐Luciferase Reporter Assay System (Promega). And the Renilla luciferase activity normalized to firefly luciferase activity. The experiments repeated independently three times, and the data are represented as mean ± SD.

### 2.9 Immunohistochemical staining

After fixation for 24 hours in 4% paraformaldehyde, the tissues were dehydrated, embedded in paraffin and sectioned perpendicularly to the direction of the muscle fibre into 5‐μm‐thick slices. Then, slices were incubated for 12 hours in a‐SMA antibody followed by secondary antibody conjugated with HRP. Subsequently, immunohistochemical staining was conducted by 3,3′‐diaminobenzidine and haematoxylin detection. All histological slices were visualized with inverted light microscopy (Olympus) and digitalized with DP72 Manager (Olympus). Digital quantification of α‐SMA positive areas was conducted with software Image‐Pro (Meida Cybernetics).

### 2.10 Immunofluorescence (IF)

C2C12 cells were seeded on glass slides in six‐well plates and incubated in DMEM until 80%‐90% confluent. After washed with PBS, cells were fixed with 4% paraformaldehyde for 10 minutes followed by permeabilization in PBS with 0.5% Trion‐X‐100 for 15 minutes and 3% BSA blockage for 30 minutes at room temperature. Immunofluorescence was then carried out with overnight Col 1 and α‐SMA antibodies incubation at 4°C and then with the corresponding secondary antibodies labelled with FITC for 45 minutes at 37°C. DAPI was added to stain the nuclei. Finally, images were taken with immunofluorescence microscope (Olympus).

### 2.11 RNA fluorescent in situ hybridization (FISH)

The Cy3‐labelled lnc‐MFAT1 probe was purchased from RiboBio. The subcellular localization of lnc‐MFAT1 was evaluated using a FISH kit (RiboBio). Confocal section images were captured using a confocal laser scanning microscope (FV1000, Olympus). The specific target probe is listed in Table S1.

### 2.12 Masson staining

After 24 hours fixation in 4% paraformaldehyde, the tissues were routinely dehydrated, embedded in paraffin and sectioned at 5‐μm thickness. Sections were incubated at 37°C overnight, dewaxed and stained with Masson trichrome according to standard procedures.^14^ Under Masson trichrome staining, the collagen fibres were stained to blue and skeletal muscle fibres to red. Digital pictures were taken using identical exposure settings for all sections. The blue area was measured as fibrotic areas with Image‐Pro (Meida Cybernetics).

### 2.13 Microarray and data analysis

In brief, 4 paired skeletal muscle fibrosis samples and normal control samples were collected. The expression of lncRNAs in skeletal muscle fibrosis was analysed using Gene Expression Hybridization Kit (Agilent technologies). Samples labelling and array hybridization were carried out following the Agilent One‐Color Microarray‐Based Gene Expression Analysis protocol (Agilent technologies) with minor modifications. Data were extracted with Feature Extraction software 10.7 (Agilent technologies). Raw data were normalized by Quantile algorithm, Gene Spring Software 12.6.1 (Agilent technologies).

lncRNAs were chosen for further data analysis after quantile normalization of the raw data. Differentially expressed lncRNAs between the two groups (fold change ≥ 2, *P* < .05) were identified from microarray data. Finally, Hierarchical Clustering and combined analysis were performed to show the differentially expressed lncRNAs between the two groups using in‐house scripts.

### 2.14 Functional group analysis of mRNAs

The differentially expressed mRNAs were mapped with the Kyoto Encyclopedia of Genes and Genomes (KEGG) analysis to investigate the biological functions using online bioinformatics tool DAVID6.8 (https://david.ncifcrf.gov/). A significant *P* value (hypergeometric *P* value) indicates a correlation between a pathway and the conditions. The recommend *P* value cut‐off is .05.

### 2.15 Statistical analysis

Data are presented as the mean ± SD. For the relative lncRNA, miRNA, mRNA, protein and luciferase activity quantification, the means of the control groups are taken as 1. Statistical analysis was performed by GraphPad Prism software (v6). To determine statistical significance of differences between groups, the Student t test was performed. A two‐side *P* values < .05 were considered statistically significant.

## 3 RESULTS

### 3.1 lnc‐MFAT1 was up‐regulated in the expression profile of lncRNAs induced by skeletal muscle fibrosis

**FIGURE 1:**
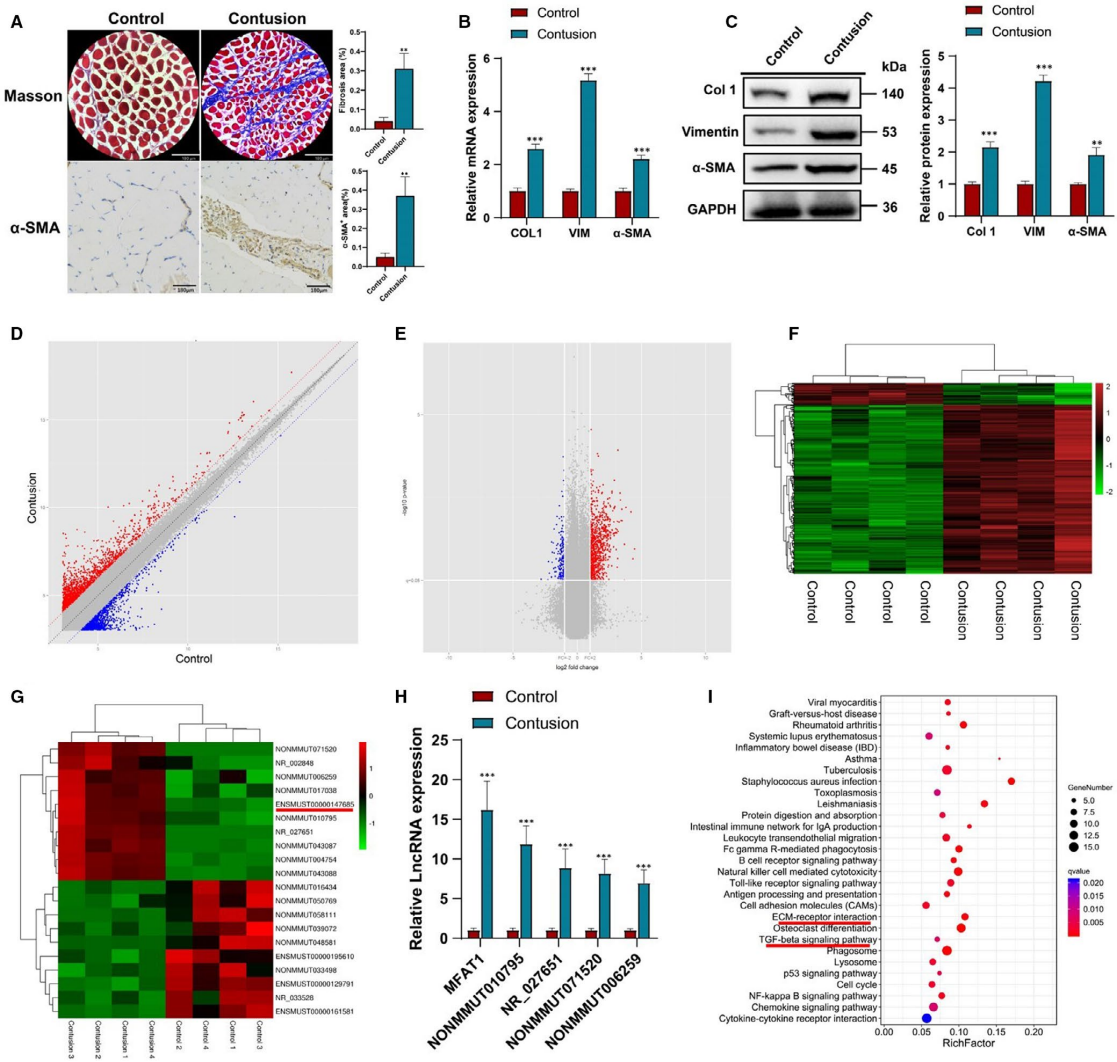
lnc‐MFAT1 was up‐regulated in the expression profile of lncRNAs induced by skeletal muscle fibrosis. A, Representative images of Masson staining and α‐SMA immunohistochemical staining showing fibrosis areas in normal control group and acute contusion group mice (n = 10). Scale bars for Masson staining and immunohistochemical staining represent 180 μm. B,C, qRT‐PCR and Western blot analysis showing the mRNA and protein levels of Col 1, VIM and α‐SMA. D,E, Volcano plot diagram and scatter diagram showing the expression correlation of these lncRNAs. F, Hierarchical clustering of differentially expressed lncRNAs in contusion and normal control group. G, Hierarchical clustering analysis of top 20 most changed lncRNAs. H, The expression level of top 5 up‐regulated lncRNAs in 10 paired acute contusion and normal control tissues by qRT‐PCR. I, KEGG pathway analysis of significant pathways. All experiments were repeated three times independently, and data are expressed as the mean ± SD. **P < .01, ***P < .001

The acute contusion‐induced mice skeletal muscle fibrosis model was established according to previous study.^7^ As shown by Masson staining, acute contusion led to a significantly larger collagen deposition area (Figure 1A). In line with it, immunohistochemical staining revealed significantly increased α‐SMA positive areas in the acute contusion group (Figure 1A). Col 1, VIM, and α‐SMA, served as fibrosis indicators, were all remarkably elevated at both mRNA and protein levels in acute contusion group (Figure 1B,C).

The lncRNA microarray was acquired from four pairs of samples between the acute contusion group and normal control group. After screening (fold change ≥ 2, *P* < .05), the levels of differentially expressed lncRNAs in the samples were displayed using volcano plot (Figure 1D) and scatter plot (Figure 1E). The expression profiling data suggested 513 differentially expressed lncRNAs in total, with 455 up‐regulated and 58 down‐regulated (Figure 1F). The top 20 mostly changed lncRNAs (fold change ≥ 2, *P* < .05) were shown in Figure 1G and Table 1. Among the top 10 up‐regulated lncRNAs, the skeletal muscle fibrosis‐associated transcript 1 (MFAT1), lncRNA‐MFAT1 (accession: ENSMUST00000147685.2, an 816 bps transcript with 3 exons and localizes in chromosome 2qA1), was the most up‐regulated lncRNA in skeletal muscle fibrosis samples according to the lncRNA microarray (Figure 1G, Table 1) and qRT‐PCR results (Figure 1H). We next examined the expression profile of lnc‐MFAT1 and found that lnc‐MFAT1 expression was detected in skeletal muscle and heart, but not in any other tissues (Figure S1). In addition, the coding ability of lnc‐MFAT1 was predicted using Ensembl Genome Browser (http://asia.ensembl.org/), prediction software and the NCBI ORF finder (https://www.ncbi.nlm.nih.gov/orffinder/); the results showed no protein‐coding capability for lnc‐MFAT1, indicating it a non‐coding RNA (Figure S2). Furthermore, KEGG pathway enrichment analysis was applied to identify the differentially expressed pathways after skeletal muscle fibrosis (Figure 1I). Among the up‐regulated signalling pathways, the TGF‐β signalling pathway (Rich factor = 0.074, *P* = .0069) and the ECM receptor interaction (Rich factor = 0.115, *P* = .0004), both of which play critical roles in the fibrosis process (Figure 1I), appeared to be most enriched pathways.

**TABLE 1:** The top 10 up‐regulated and down‐regulated lncRNAs in lncRNA microarray

Track_id	Gene_Name	Gene_Type	log2FC	Fold_Change	*p*_value
Up‐regulated lncRNA					
lnc‐MFAT1	ENSMUSG00000086109	lincRNA	4.461675963	22.03425118	.01003686
NONMMUT010795	NONMMUG006804	lincRNA	4.335979531	20.19574598	.01664927
NR_027651	Meg3	lincRNA	4.187403914	18.21940467	.00149459
NONMMUT071520	NONMMUG044313	lincRNA	3.792151656	13.85324122	.00118783
NONMMUT006259	NONMMUG004024	lincRNA	3.679796532	12.81531053	.04960561
NR_002848	6430411K18Rik	lincRNA	3.678257743	12.80164891	.00790285
NONMMUT043087	NONMMUG026611	intronic_antisense	3.496027981	11.28260251	.00236225
NONMMUT004754	NONMMUG003054	intronic_antisense	3.474749184	11.1174128	.00129487
NONMMUT017038	NONMMUG010713	lincRNA	3.414837381	10.66518718	.00233095
NONMMUT043088	NONMMUG026611	lincRNA	3.359659288	10.26498268	.00081285
Down‐regulated lncRNAs					
ENSMUST00000195610	ENSMUSG00000102238	intronic_antisense	−1.835418771	0.280210171	.02725218
ENSMUST00000129791	ENSMUSG00000087662	lincRNA	−1.873329596	0.272942773	.00733075
NONMMUT033498	NONMMUG020611	lincRNA	−1.939866397	0.260640576	.01189769
NR_033528	Olfr75‐ps1	lincRNA	−1.958483283	0.257298815	.01989215
ENSMUST00000161581	ENSMUSG00000089699	intronic_antisense	−1.998685326	0.25022792	.0070162
NONMMUT016434	NONMMUG010329	lincRNA	−2.014599937	0.247482782	.04239784
NONMMUT050769	NONMMUG031471	intronic_antisense	−2.171127882	0.222037016	.03462806
NONMMUT058111	NONMMUG036089	lincRNA	−2.241445648	0.211474315	.01914533
NONMMUT039072	NONMMUG024130	lincRNA	−2.288072109	0.20474894	.04900814
NONMMUT048581	NONMMUG030150	lincRNA	−2.812201456	0.142378039	.04764284

### 3.2 lnc‐MFAT1 was up‐regulated in C2C12 cells stimulated with TGF‐β1

**FIGURE 2:**
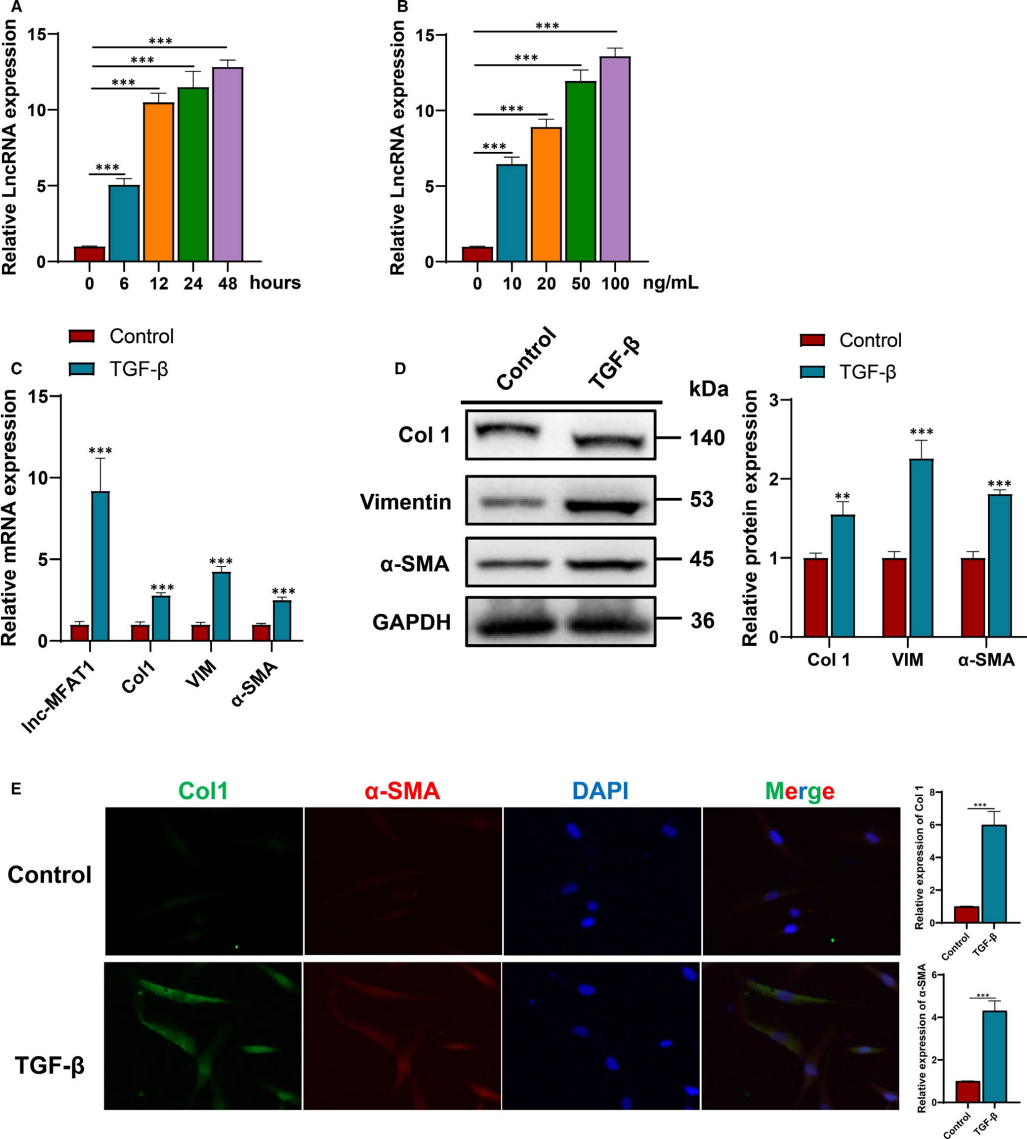
lnc‐MFAT1 was up‐regulated in C2C12 cells stimulated with TGF‐β1. A,B, qRT‐PCR showing the lnc‐MFAT1 expression levels in C2C12 cells treated with different concentrations of TGF‐β at different times. C2C12 cells were cultured in serum‐free DMEM and treated with TGF‐β (10 ng/mL for 0, 6, 12, 24 and 48 h, or 0, 10, 20, 50 and 100 ng/mL for 12 h). C,D, qRT‐PCR and Western blot analysis showing the mRNA and protein levels of Col 1, VIM and α‐SMA. E, Representative images of immunofluorescence staining of Col 1‐positive cells (green) and α‐SMA ‐positive cells (red) after cultured with or without TGF‐β (10 ng/mL) in serum‐free DMEM for 12 h. Nuclei are counterstained with DAPI (blue). All experiments were repeated three times independently, and data are expressed as the mean ± SD. **P < .01, ***P < .001

TGF‐β1 stimulation induces the activation of C2C12 myoblasts into fibroblasts in vitro, which has been well‐established and widely validated in previous studies.^7^, ^15^, ^16^ Consistent with previous studies, TGF‐β1 stimulation remarkably up‐regulated lnc‐MFAT1 expression level (Figure 2A,B), correlating with an increase of Col 1, VIM and α‐SMA at both mRNA and protein levels (TGF‐β1, 10 ng/mL, 12 hours) (Figure 2C,D). Additionally, an increased expression of α‐SMA (4.3‐fold) was detected after incubated with TGF‐β, suggesting that fibroblasts differentiated into myofibroblasts (Figure 2E). Consistent with this, an increased expression of Col 1 (6.0‐fold) was confirmed in the TGF‐β1 stimulation group, representing more extracellular matrix formation (Figure 2E).

### 3.3 Knockdown of lnc‐MFAT1 attenuated the fibrosis of C2C12 cells

**FIGURE 3:**
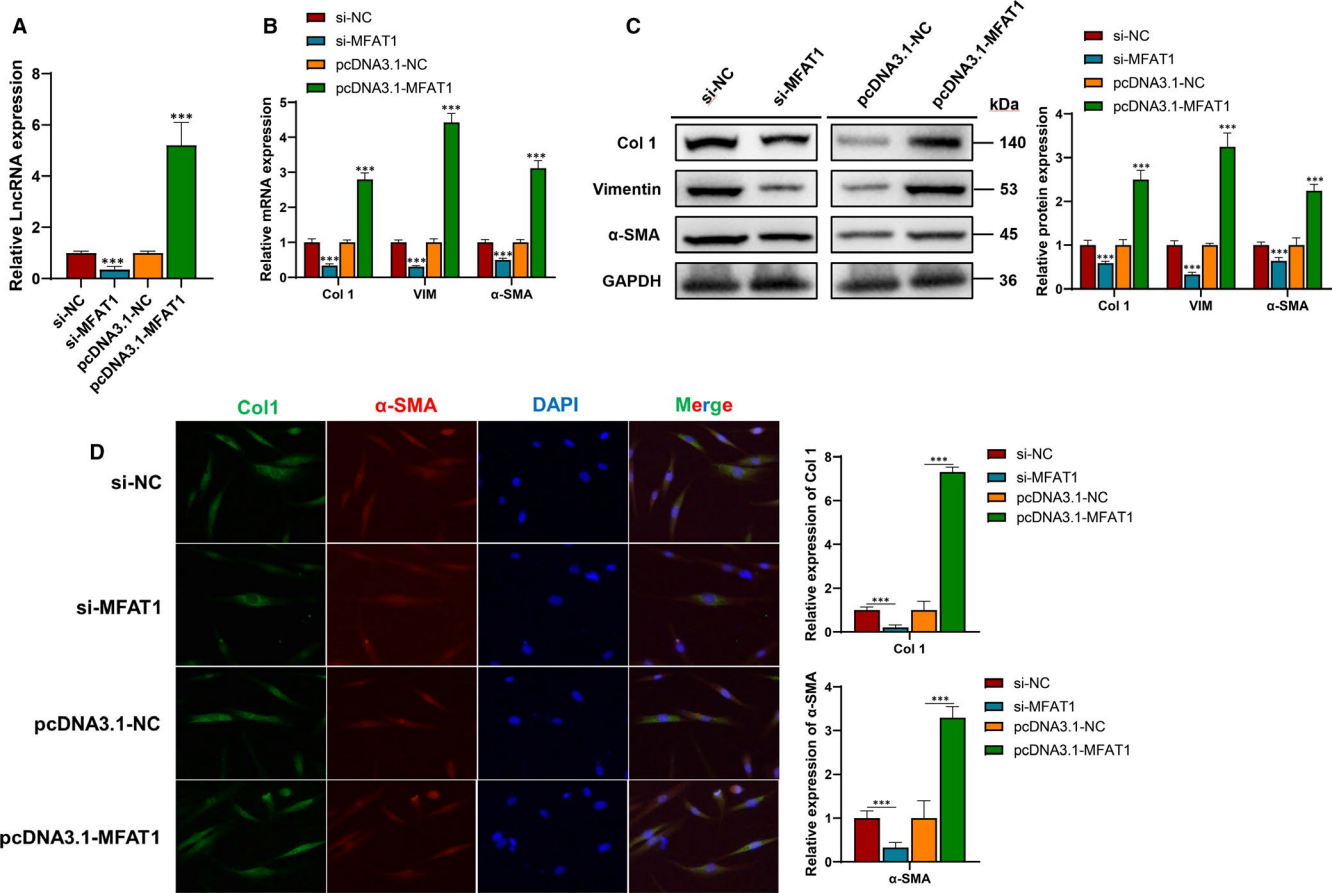
Knockdown of lnc‐MFAT1 attenuated the fibrosis of C2C12 cells. A, qRT‐PCR showing the level of lnc‐MFAT1in C2C12 cells transfected with si‐MFAT1 or pcDNA3.1‐MFAT1, respectively. B,C, qRT‐PCR and Western blot analysis showing the mRNA and protein levels of Col 1, VIM and α‐SMA in C2C12 cells transfected with si‐MFAT1 or pcDNA3.1‐MFAT1, respectively. D, Representative images of immunofluorescence staining of Col 1‐positive cells (green) and α‐SMA ‐positive cells (red) after transfected with si‐MFAT1 or pcDNA3.1‐MFAT1 in TGF‐β (10 ng/mL) stimulated C2C12 cells. Nuclei are counterstained with DAPI (blue). All experiments were repeated three times independently, and data are expressed as the mean ± SD. ***P < .001

To investigate the role of lnc‐MFAT1 in C2C12 cells, lnc‐MFAT1 was knocked down by transfecting MFAT1‐siRNA (si‐MFAT) and overexpressed by transfecting pcDNA3.1‐MFAT1 in C2C12 cells. Then, compared with the negative control (si‐NC), the expression level of MFAT1 remarkably decreased or increased, respectively (Figure 3A). After transfecting si‐MFAT1, the expression of Col1, VIM and α‐SMA was significant down‐regulated compared with si‐NC group (Figure 3B,C). Immunofluorescence staining indicated that α‐SMA and Col 1 were remarkably down‐regulated in si‐MFAT1 group versus si‐NC group (Figure 3D). Meanwhile, the opposite effects of lnc‐MFAT1 overexpression (pcDNA3.1‐MFAT1) were also displayed (Figure 3A‐D).

### 3.4 lnc‐MFAT1 acted as a ceRNA to sponge miR‐135a‐5p in C2C12 cells

**FIGURE 4:**
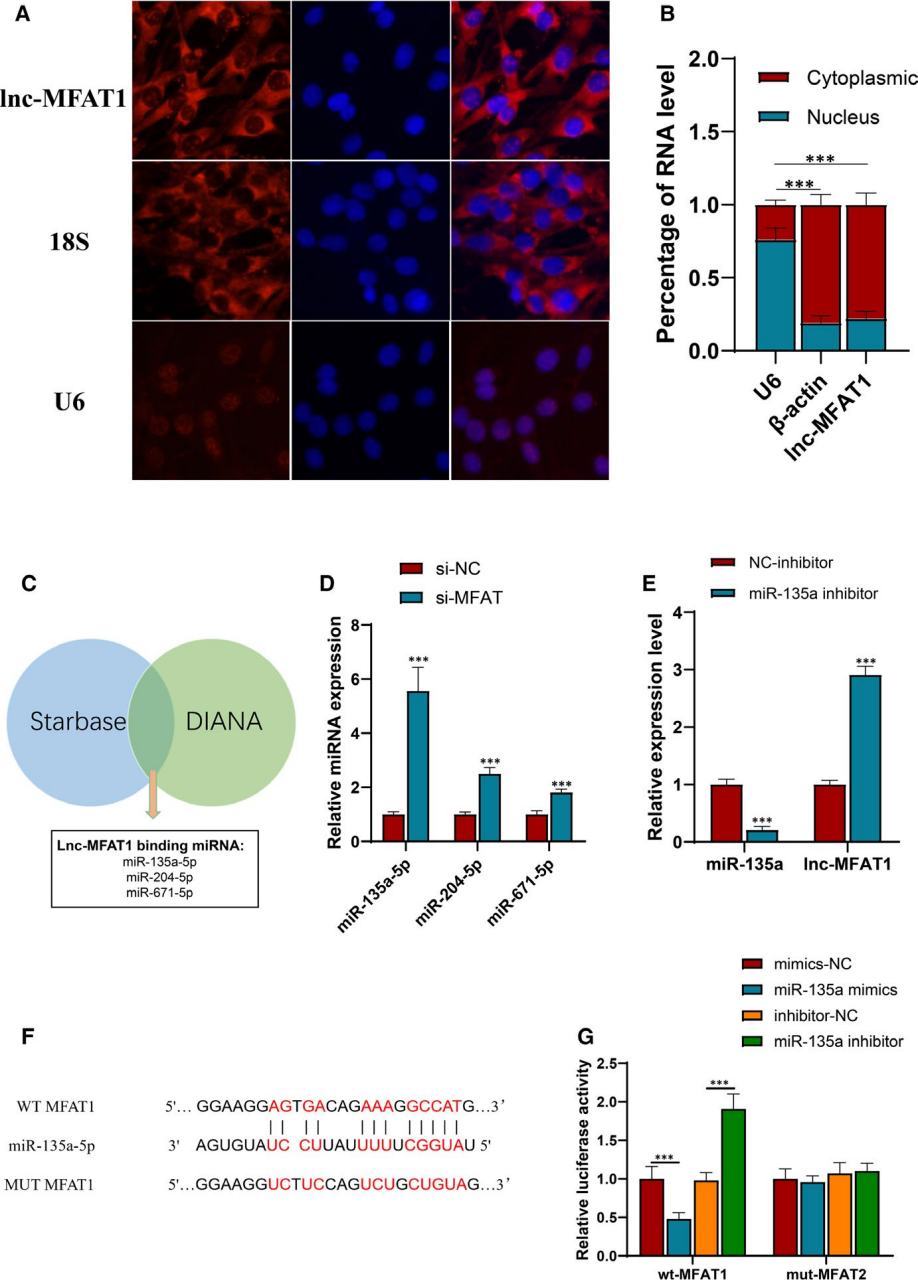
lnc‐MFAT1 acted as a ceRNA to sponge miR‐135a‐5p in C2C12 cells. A,B Subcellular localization of lnc‐MFAT1 in C2C12 cells was detected by FISH and qRT‐PCR. C, Starbase v3.0 and DIANA databases predict miRNAs binding to MFAT1. D, qRT‐PCR showing the levels of miRNAs after lnc‐MFAT1 knockdown or overexpression. E, qRT‐PCR showing the levels of miR‐135a and lnc‐MFAT1 after transfected with miR‐135a inhibitor. F, Schematic diagram of the predicted miR‐135a binding sites in lnc‐MFAT1 3′‐UTR. G, Dual‐luciferase reported assay was conducted by cotransfection with miR‐135a mimics/inhibitors and luciferase plasmids containing WT/MUT lnc‐MFAT1 3′‐UTR in HEK293T cells. All experiments were repeated three times independently, and data are expressed as the mean ± SD. ***P < .001

Given the association between lncRNA function and its subcellular distribution, FISH and subcellular fractionation assays were used to identify the lnc‐MFAT1 as a cytoplasmic‐localized lncRNA (Figure 4A,B), which could be a competing endogenous RNA (ceRNA) to regulate miRNAs. To seek miRNAs interacted with lnc‐MFAT1, the miRNA prediction tools (Starbase v3.0 and DIANA tools) were performed (Figure 4C) and three high related miRNAs (mmu‐miR‐135a‐5p, mmu‐miR‐204‐5p and mmu‐miR‐671‐5p) were found. Furtherly, qRT‐PCR showed that the miR‐135a‐5p(miR‐135a) significantly increased after lnc‐MFAT1 knockdown (Figure 4D). To further verify the association between lnc‐MFAT1 and miR‐135a, miR‐135a inhibitor and its negative control were transfected. As shown in Figure 4E, miR‐135a inhibitor resulted in a significant up‐regulation of lnc‐MFAT1 level.

The binding sites between lnc‐MFAT1 3′‐UTR and miR‐135a were predicted using bioinformatics analysis (Figure 4F). After that, lnc‐MFAT1 3′‐UTR wild‐type or mutated binding sites were cloned into pSI‐Check2 vector and luciferase activity was detected after cotransfection of miR‐135a and luciferase plasmids. As predicted, cotransfection of lnc‐MFAT1 3′‐UTR wild‐type luciferase plasmids and miR‐135a mimics significantly attenuated the luciferase activity, whereas miR‐135a inhibitor increased the luciferase activity. Yet, cotransfection of the lnc‐MFAT1 3′‐UTR mutant luciferase plasmids and miR‐135a mimics or inhibitor had no effect on the luciferase activity (Figure 4G).

### 3.5 miR‐135a inhibition increased the expression of fibrosis‐related proteins through Tgfbr2/Smad4 signalling

**FIGURE 5:**
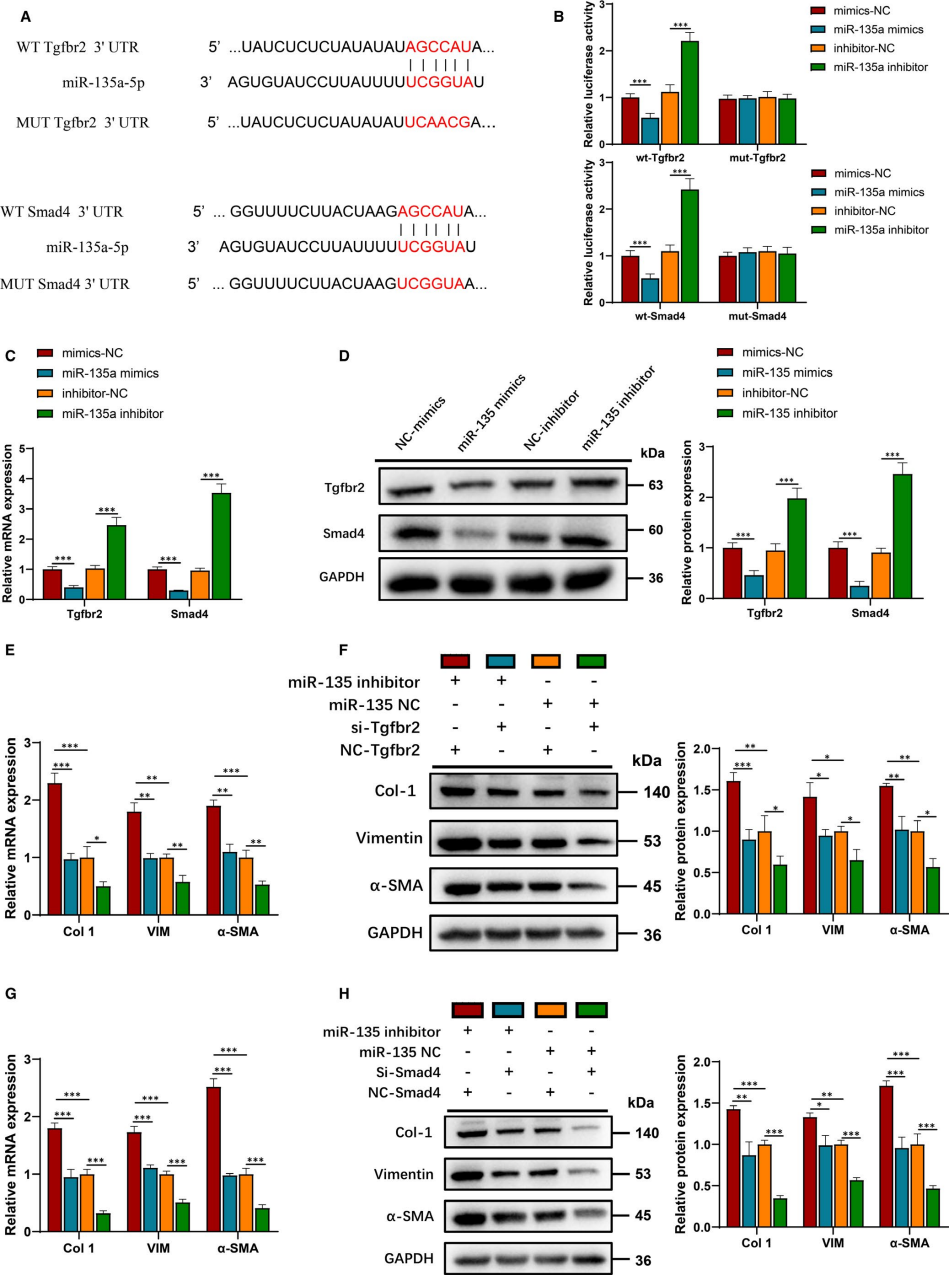
miR‐135a inhibition increased the expression of fibrosis‐related proteins through Tgfbr2/Smad4 signalling. A, Schematic diagram of the predicted miR‐135a binding sites in Tgfbr2 3′‐UTR or Smad4 3′‐UTR, respectively. B, Dual‐luciferase reported assay was conducted by cotransfection with miR‐135a mimics/inhibitors and luciferase plasmids containing WT/MUT Tgfbr2 3′‐UTR or WT/MUT Smad4 3′‐UTR in HEK293T cells. C,D, qRT‐PCR and Western blot analysis showing the mRNA and protein levels of Tgfbr2 and Smad4 in C2C12 cells transfected with miR‐135a mimics/inhibitors/negative control, respectively. E‐H, qRT‐PCR and Western blot analysis showing the mRNA and protein levels of Col 1, VIM and α‐SMA. C2C12 cells transfected with miR‐135a inhibitor, si‐Tgfbr2, si‐Smad4 or their negative control. All experiments were repeated three times independently, and data are expressed as the mean ± SD. *P < .05, **P < .01, ***P < .001

Combined with previous studies^6^, ^7^ and bioinformatics analysis by TargetScan (http://www.targetscan.org/vert_72/), Tgfbr2 and Smad4 were selected for miR‐135a candidate prediction. The predicted binding sites between Tgfbr2 3′‐UTR, Smad4 3′‐UTR and miR‐135a were shown in Figure 5A. The results showed that the luciferase activities remarkably attenuated by cotransfection of Tgfbr2 3′‐UTR, Smad4 3′‐UTR wild‐type luciferase plasmids and miR‐135a mimics and increased by miR‐135a inhibitor. By contrast, cotransfection of the mutant luciferase plasmids and miR‐135a mimics or inhibitor had no effect on the luciferase activity (Figure 5B).

A decrease in Col1, VIM and α‐SMA protein expression was noticeable after transfecting of with miR‐135a mimics (Figure S3). Meanwhile, the opposite effects of miR‐135a inhibitor were also displayed (Figure S3). qRT‐PCR and Western blot analysis demonstrated that miR‐135a mimics transfection attenuated Tgfbr2 and Smad4 expression (Figure 5C,D), whereas miR‐135a inhibitor transfection showed opposite trend (Figure 5C,D). In addition, silencing Tgfbr2 or Smad4 reversed the promoting effect of miR‐135a inhibitor on the mRNA and protein expression of Col1, VIM and α‐SMA (Figure 5E,F). Thus, miR‐135a inhibition increased the expression levels of fibrosis‐related proteins through the Tgfbr2/Smad4 axis.

### 3.6 lnc‐MFAT1 promoted Tgfbr2/Smad4 expression and enhanced the fibrosis of C2C12 cells by acting as a miR‐135a sponge

**FIGURE 6:**
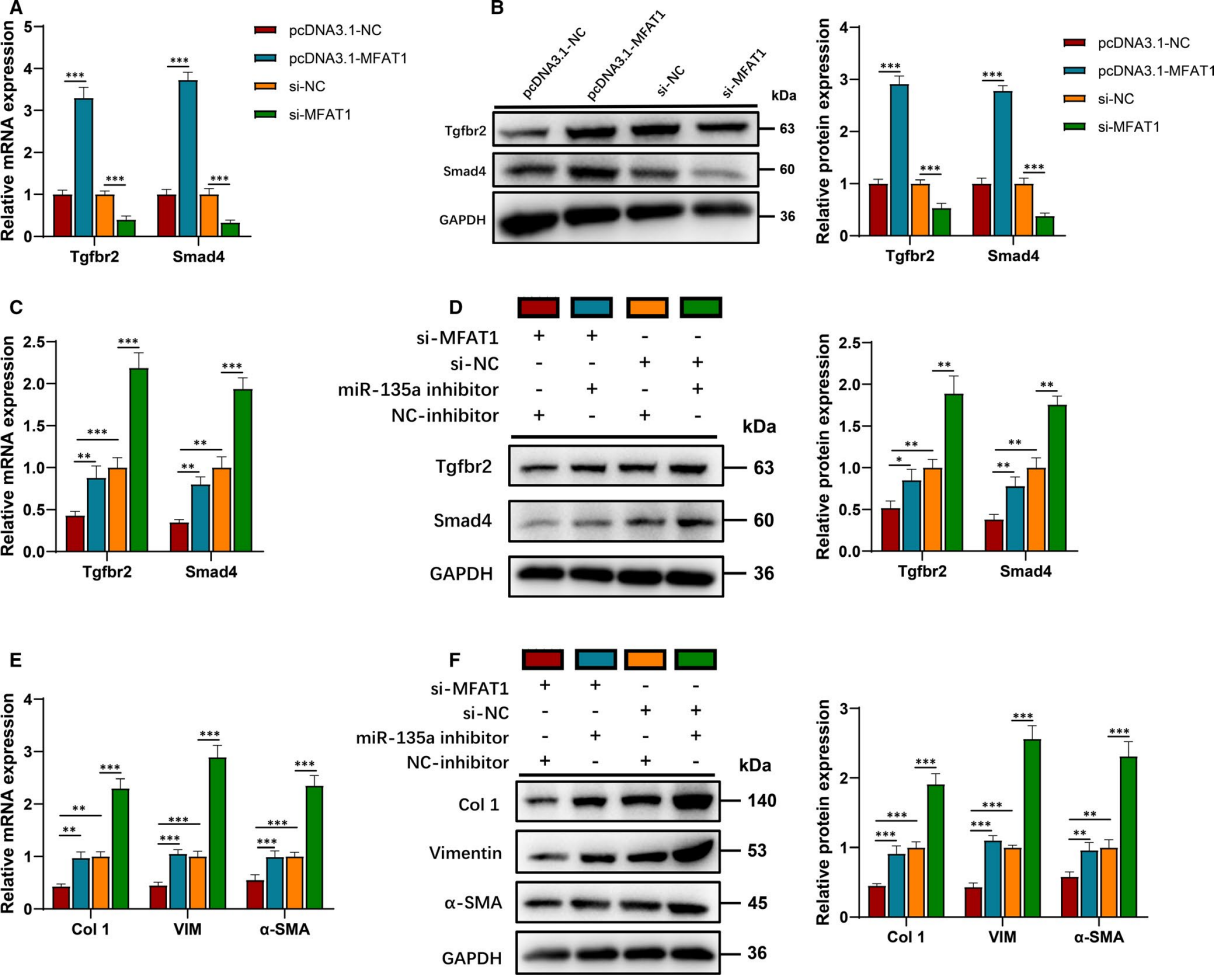
lnc‐MFAT1 promoted Tgfbr2/Smad4 expression and the fibrosis of C2C12 cells by acting as a miR‐135a sponge. A,B, qRT‐PCR and Western blot analysis showing the mRNA and protein levels of Tgfbr2/Smad4. C2C12 cells were transfected with MFAT1‐pcDNA3.1 or MFAT1‐shRNA, respectively. C,D, qRT‐PCR and Western blot analysis showing the mRNA and protein levels of Tgfbr2/Smad4. C2C12 cells were transfected with MFAT1‐shRNA, miR‐135a inhibitor or their negative control. G‐I, qRT‐PCR and Western blot analysis showing the mRNA and protein levels of Col 1, VIM and α‐SMA. C2C12 cells were transfected with MFAT1‐shRNA, miR‐135a inhibitor or their negative control. All experiments were repeated three times independently, and data are expressed as the mean ± SD. *P < .05, **P < .01, ***P < .001

The qRT‐PCR and Western blot analysis demonstrated that the overexpression and knockdown of lnc‐MFAT1 increased and decreased Tgfbr2/Smad4 expression, respectively (Figure 6A,B). Then, after cotransfecting si‐MFAT1 and miR‐135a inhibitor into C2C12 cells, qRT‐PCR and Western blot analysis showed that miR‐135a inhibitor could restore the expression of Tgfbr2/Smad4 (Figure 6C,D) and fibrosis markers (Figure 6E,F), which could be down‐regulated by lnc‐MFAT1 silencing.

### 3.7 lnc‐MFAT1 knockdown inhibited fibrosis, up‐regulated miR‐135a level and blocked TGF‐β/Smads pathway activation in vivo

**FIGURE 7:**
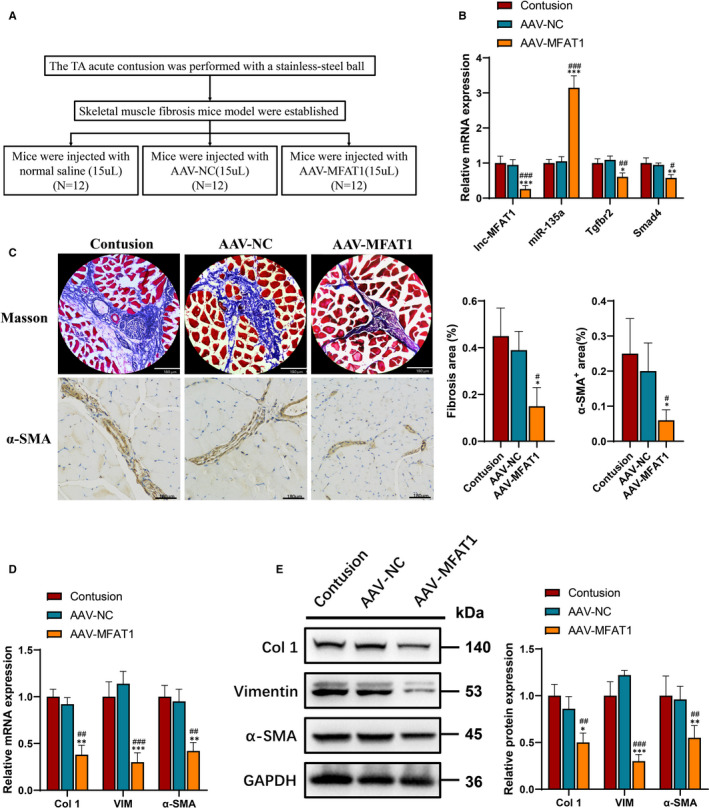
lnc‐MFAT1 knockdown inhibited fibrosis, up‐regulated miR‐135a level and blocked TGF‐β/Smads pathway activation in vivo. A, Establishment procedure of MFAT1 knockdown in skeletal muscle fibrosis mice models (AAV‐MFAT1 group = 12, AVV‐NC group = 12, contusion group = 12). B, qRT‐PCR analysis showing the mRNA levels of lnc‐MFAT1, miR‐135a and Tgfbr2/Smad4. C, Representative images of Masson staining and α‐SMA immunohistochemical staining showing fibrosis areas in contusion, AVV‐NC and AAV‐MFAT1 group mice (n = 12). Scale bars for Masson staining and immunohistochemical staining represent 180 μm. D,E, qRT‐PCR and Western blot analysis showing the mRNA and protein levels of Col I, VIM and α‐SMA. All experiments were repeated three times independently, and data are expressed as the mean ± SD. */#*P* < .05, **/##*P* < .01,***/###*P* < .001,* compared with contusion group, # compared with AAV‐NC group

An lnc‐MFAT1 knockdown mice model was built by adeno‐associated virus (AAV) injection to further study the effect of lnc‐MFAT1 on skeletal muscle fibrosis in vivo. The establishment procedure is shown in Figure 7A. The efficiency of AAV‐MFAT1 was detected in skeletal muscle fibrosis tissues by qRT‐PCR, which indicated that lnc‐MFAT1 was successfully down‐regulated after AAV‐MFAT1 injection (Figure 7B). The expressions of miR‐135a, Tgfbr2 and Smad4 were detected in skeletal muscle fibrosis tissues to validate the participation of the lnc‐MFAT1‐miR‐135a‐Tgfbr2/Smad4 axis in skeletal muscle fibrosis (Figure 7B).

Masson staining and immunofluorescence staining results showed that lnc‐MFAT1 knockdown inhibited skeletal muscle fibrosis development compared with that in the contusion and AAV‐NC group (Figure 7C). In addition, fibrosis‐related genes were detected in the model. qRT‐PCR and Western blot analysis demonstrated that Col 1, VIM and α‐SMA were down‐regulated in the AAV‐MFAT1 skeletal muscle fibrosis mice model (Figure 7D,E). These results demonstrated that lnc‐MFAT1 knockdown inhibited skeletal muscle fibrosis via the miR‐135a‐Tgfbr2/Smad4 axis in vivo.

## 4 DISCUSSION

In this study, lnc‐MFAT1 was confirmed to be substantially up‐regulated in skeletal muscle fibrosis in vivo and in vitro, and its silence could attenuate the skeletal muscle fibrosis following an acute contusion in vivo and mitigate the proliferation of TGFβ‐induced C2C12 cells into myofibroblasts. Furthermore, it was predicted by the miRNA target prediction bioinformatics and confirmed by dual‐luciferase reporter assays that miR‐135a could simultaneously target lnc‐MFAT1 and Tgfbr2/Smad4. Silencing lnc‐MFAT1 attenuated, whereas inhibiting miR‐135a promoted, the expression of Tgfbr2, Smad4 and ECM markers. In addition, miR‐135a inhibition could remarkably restore the effects of lnc‐MFAT1 silence on Tgfbr2, Smad4 and ECM markers. In summary, our study verified that lnc‐MFAT1 could regulate skeletal muscle fibrosis by acting as a ceRNA to sponge miR‐135a through the miR‐135a‐Tgfbr2/Smad4 axis.

Initially, the current study revealed that lnc‐MFAT1 was essential for maintaining skeletal muscle fibrosis both in vivo and in vitro. Multiple evidence showed that lncRNAs are aberrantly expressed in various organ fibrosis.^9^, ^17^, ^18^, ^19^ lncRNA Meg3 was found to be mostly up‐regulated in cardiac fibrosis.^20^ Inhibition of Meg3 prevented cardiac MMP‐2 induction, resulting in cardiac fibrosis decreasing and diastolic performance improving in vivo and in vitro.^20^ Moreover, lncRNA ITPF was up‐regulated in idiopathic pulmonary fibrosis, which could promote pulmonary fibrosis by targeting hnRNP‐L depending on its host gene ITGBL1.^19^ Consistent with previously reported lncRNAs, lnc‐MFAT1 was shown to be up‐regulated in skeletal muscle fibrosis in our study (Figures 1 and 2). Additionally, lnc‐MFAT1 silencing attenuated the expression of the fibrotic indicators and immunohistochemically positive area both in vitro and in vivo (Figures 3 and 7).

In addition, the current study showed that lnc‐MFAT1 could regulate skeletal muscle fibrosis by acting as a miR‐135a ceRNA. It have been proposed that the biological function of lncRNA was closely related to its subcellular location.^21^ Typically, lncRNAs localized in the cytoplasm regulate signalling pathways and mRNA stability or translation, whereas lncRNAs localized in the nucleus participate in RNA processing, transcriptional regulation and chromatin interactions. The lncRNAs localized in the cytoplasm were suggested to regulate the target mRNA by sponging to miRNAs. For example, lnc Kcnq1ot1 alleviates fibrosis through sponging miR‐214‐3p to regulate TGF‐β1/Smads pathway in high glucose‐treated cardiac fibroblasts.^8^ Additionally, lncRNA PFAR promotes lung fibroblast activation and fibrosis by targeting miR‐138 to regulate the YAP1‐Twist axis.^22^ In the current study, we demonstrated that lnc‐MFAT1 predominately localized in the cytoplasm (Figure 4). Subsequent bioinformatics analysis and dual‐luciferase reporter assays showed that both lnc‐MFAT1 and Tgfbr2/Smad4 contain binding sites of miR‐135a (Figure 4), whose anti‐fibrotic effects were reported in pulmonary^23^ and cardiac^24^ fibrosis. In current study, miR‐135a up‐regulation and down‐regulation could, respectively, attenuate and promote the level of fibrotic indicators, indicating the inhibitory effect of miR‐135a on skeletal muscle fibrosis (Figure 6).

Further, Tgfbr2/Smad4 was proved to be target genes of miR‐135a and played an important role in mediating the fibrosis function of lnc‐MFAT1/miR‐135a. TGF‐β exerts its effects by binding to Tgfbr1/2, leading to phosphorylation of Smad2/3. Subsequently, activated Smad2/3 binds to Smad4 and translocates into the nucleus, which initiates TGF‐β signalling pathway.^25^ Previous studies discovered that several lncRNAs interacted with TGF‐β/Smads in the promotion of liver^9^, ^26^ and atrial fibrosis.^27^ In this study, the profibrotic effect of miR‐135a inhibitor transfection was attenuated by Tgfbr2 or Smad4 silencing, suggesting that the anti‐fibrotic role of miR‐135a was mediated by Tgfbr2/Smad4 (Figure 5). Additionally, lnc‐MFAT1 silencing suppressed the expression of Tgfbr2/Smad4, and miR‐135a inhibition could restore the expression (Figure 6). These results suggest that Tgfbr2/Smad4 is crucial in mediating the fibrosis function of lnc‐MFAT1/miR‐135a.

**FIGURE 8:**
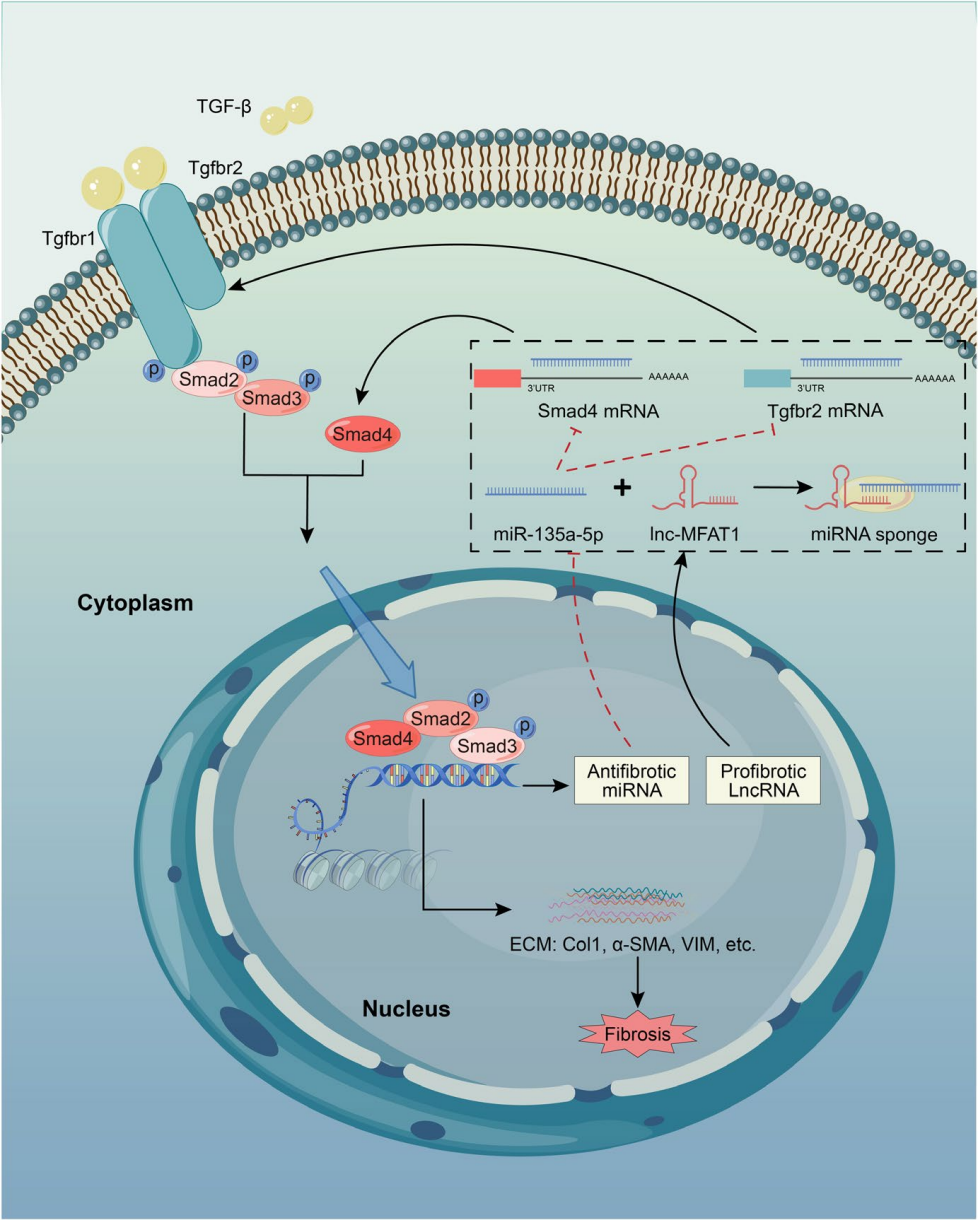
Schematic illustration of lnc‐MAFAT1‐miR‐135a‐Tgfbr2/Smad4 axis in skeletal muscle fibrosis

In summary, the newly identified lnc‐MFAT1 was found to be up‐regulated in skeletal muscle fibrosis tissues. Through integrating in vivo and in vitro experiments, we illustrated that lnc‐MFAT1 acts as an miR‐135a sponge to regulate Tgfbr2/Smad4, resulting in skeletal muscle fibrosis (Figure 8). This study provides novel insights into the regulation of skeletal muscle fibrosis by lncRNAs and suggests lnc‐MFAT1 as a promising therapeutic target.

## CONFLICT OF INTEREST

The authors declare no competing financial interests.

## AUTHOR CONTRIBUTION

**Jinrong Lin:** Conceptualization (lead); Project administration (lead); Writing‐review & editing (lead). **Xiaobao Yang:** Conceptualization (equal); Project administration (supporting). **Shaohua Liu:** Methodology (equal); Project administration (equal); Writing‐review & editing (equal). **Zhiwen Luo:** Methodology (lead). **Qingyan Chen:** Data curation (equal); Methodology (equal); Writing‐review & editing (equal). **Yaying Sun:** Conceptualization (equal); Methodology (equal). **Zheci Ding:** Methodology (equal); Writing‐original draft (supporting). **Jiwu Chen:** Conceptualization (lead); Writing‐review & editing (lead).

## Supporting information

Supplementary Material
