# Supplementary material for: Long non‐coding RNA MFAT1 promotes skeletal muscle fibrosis by modulating the miR‐135a‐5p‐Tgfbr2/Smad4 axis as a ceRNA
Source: J Cell Mol Med. 2021 Apr 9;25(9):4420–33. doi: 10.1111/jcmm.16508 (PMC8093971; doi:10.1111/jcmm.16508)
Supplement: Supplementary file 1 — Supplementary Material [file JCMM-25-4420-s001.docx]

**Table S1 Antibodies used in the experiment.**

| **Gene** | **Primer** | **Sequence(5′-3′)** |
| --- | --- | --- |
| **Primers for qRT-PCR** | | |
| Collagen1 | forward | GCTCCTCTTAGGGGCCACT |
|  | reverse | CCACGTCTCACCATTGGGG |
| Vimentin | forward | CGGCTGCGAGAGAAATTGC |
|  | reverse | CCACTTTCCGTTCAAGGTCAAG |
| α-SMA | forward | GTCCCAGACATCAGGGAGTAA |
|  | reverse | TCGGATACTTCAGCGTCAGGA |
| TGFBR2 | forward | CCGCTGCATATCGTCCTGTG |
|  | reverse | AGTGGATGGATGGTCCTATTACA |
| Smad4 | forward | CGGCCGTGGCAGGGAACA |
|  | reverse | CTGCAGAGCTCGGTGAAGGTGAAT |
| GAPDH | forward | GGCATGGACTGTGGTCATGAG |
|  | reverse | TGCACCACCAACTGCTTAGC |
| U6 | forward | ATTCGTGAAGCGTTCCATAT |
|  | reverse | CTCAAGTGTCGTGGAGTCGGCAA |
| 18S | forward | ACCGCAGCTAGGAATAATGGA |
|  | reverse | CAAATGCTTTCGCTCTGGTC |
| lnc-MFAT1 | forward | GAACAGCAGCGGTCTCTAACTTCC |
|  | reverse | CACACACTGGCACTTACACAGGAG |
| NONMMUT010795.2 | forward | GCTTCTGCCAGTCTGCGTGAG |
|  | reverse | GATCCCTCTGTCCCAACCTCCTG |
| NONMMUT071520.2 | forward | ACTCTGTGGCTGTGTGCTTGTG |
|  | reverse | GTCCTGAACTACTGTGGCTTTCCC |
| NONMMUT006259.2 | forward | AGCCAGCCTCTTCCAGTCTACAG |
|  | reverse | TGTCAGCCCATCTATCCTCTCCAC |
| NR_027651 | forward | CATCATCGGCTCACACCAGTCTTC |
|  | reverse | GAATCCGTGCTCCTTGGCTCAG |
| miR-135a-5p | forward | UAUGGCUUUUUAUUCCUAUGUGA |
|  | reverse | AGUGUAUCCUUAUUUUUCGGUAU |
| **Sequences for gene knockdown** |  | **Sequence(5′-3′)** |
| si-lnc-MFAT1 |  | CCGGCUGACAUCUACUUUA |
|  |  | UAAAGUAGAUGUCAGCCGG |
| si-Tgfbr2 |  | GCCAACAACAUCAACCACATT |
|  |  | UGUGGUUGAUGUUGUUGGCTT |
| si-Smad4 |  | CACCAGGAAUUGAUCUCUCAGGAUU |
|  |  | AAUCCUGAGAGAUCAAUUCCUGGUG |
| miR-135a-5p mimics |  | UAUGGCUUUUUAUUCCUAUGUGA |
| miR-135a-5p inhibitor |  | UUCACAUAGGAAUAAAAAGCCAUA |
|  |  | TAACACGTCTATACGCCCA |
| **Sequences of probes for lnc-MFAT1 FISH** | |  |
| 5’-cy3-GGGTCTTGCTTGGTGCCTTTCCCCTTGTCT-cy3-3’ | | |

**Table S2 Antibodies used in the experiment.**

| **Antibody** | **Source** | **Catalog. #** | **Host species** | **Applications** | **Mol.Wt(KD)** |
| --- | --- | --- | --- | --- | --- |
| Collagen1 | Affinity | [AF7001](http://www.affbiotech.cn/goods-2057-AF7001-Collagen+I+Antibody.html) | Rabbit mAb | WB (1:1000) | 139 |
|  |  |  |  | IF (1:200) |  |
| Vimentin | Affinity | [AF7013](http://www.affbiotech.cn/goods-2066-AF7013-Vimentin+Antibody.html) | Rabbit mAb | WB (1:1000) | 53 |
| α-SMA | Affinity | AF1032 | Rabbit mAb | WB (1:1000) | 45 |
|  |  |  |  | IHC (1:50) |  |
| α-SMA | Abcam | ab7817 | Mouse mAb | IF (1:200) | 45 |
| TGFBR2 | Abcam | ab186838 | Rabbit mAb | WB (1:1000) | 65 |
| Smad4 | Affinity | AF0369 | Rabbit mAb | WB (1:1000) | 60 |
| GAPDH | Affinity | AF7021 | Rabbit mAb | WB (1:1000) | 37 |
| Goat anti-rabbit IgG | Affinity | S0001 | Goat mAb | WB (1:5000) |  |
| Donkey anti-rabbit IgG | Invitrogen | A21206 | Donkey mAb | IF (1:1000) |  |
| Donkey anti-mouse IgG | Invitrogen | A32744 | Donkey mAb | IF (1:1000) |  |

**
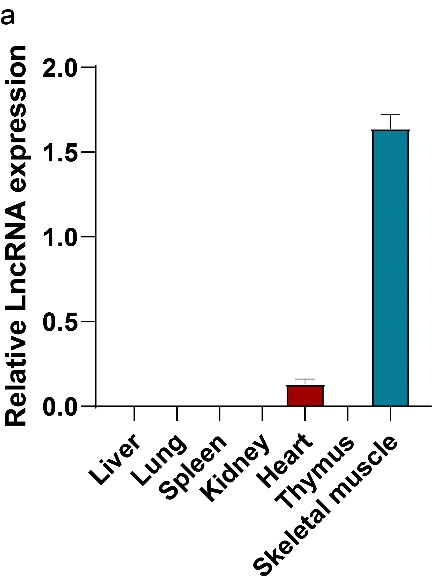
**

**Figure S1. Expression profile of lnc-MFAT1 in different tissues.**

**a** lnc-MFAT1 expression was only detected in skeletal muscles and heart, but not in any other tissues examined, including liver, lung, spleen, kidney, and thymus.

**
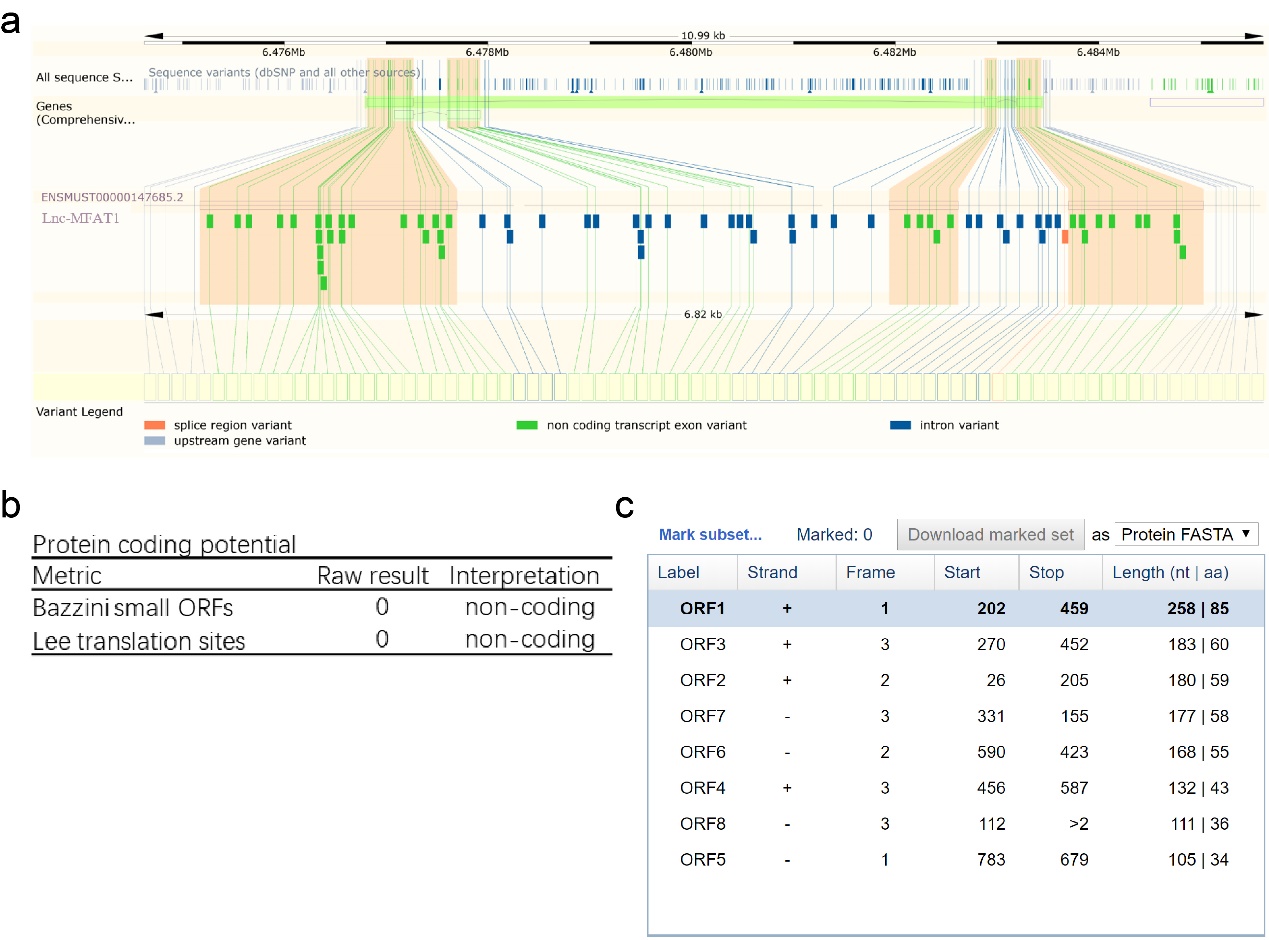
**

**Figure S2. The non-coding nature of lnc-MFAT1 was confirmed by coding-potential analysis.**

**a** lnc-MFAT1 is located on chromosome 2 in mice and composed of four exons with a full length of 2519 bp without protein-coding potential. **b** The coding potential of lnc-MFAT1 predicted in two software, and results showed that lnc-MFAT1 didn’t have any coding potential. **c** ORF Finder software prediction (https://www.ncbi.nlm.nih.gov/orffinder/) for the protein-coding potential of lnc-MFAT1.


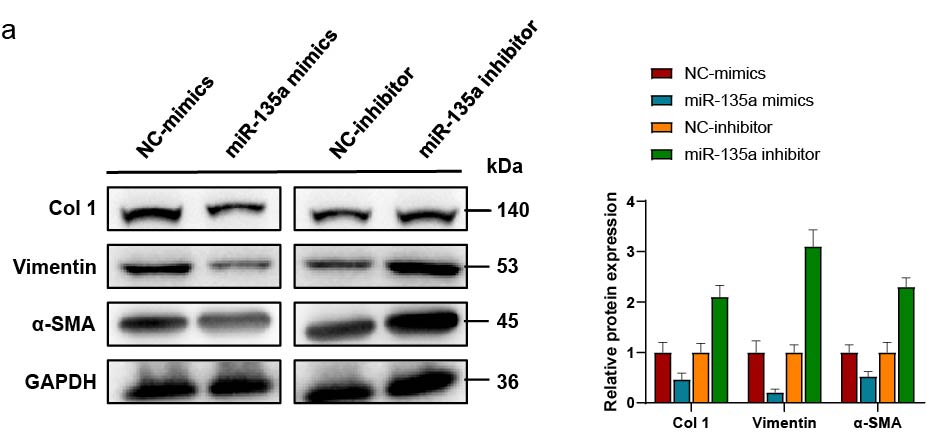


**Figure S3. Effects of miR-135a knockdown or overexpression for TGFβ1-induced fibrosis *in vitro.* a** The protein expression levels of Col 1, VIM, α-SMA in C2C12 cells treated with miR-135a mimics and miR-135a inhibitor, respectively. All data are presented as the mean ± SD of three independent experiments. ***p < 0.001.
